# Gastric Extramural Tumor Caused by Mycobacteria Infection With Immunoreconstitution Inflammatory Syndrome

**DOI:** 10.7759/cureus.29428

**Published:** 2022-09-21

**Authors:** Shu Kojima, Takeharu Asano, Takehiro Ishii, Takahiko Fukuchi, Hirosato Mashima

**Affiliations:** 1 Department of Gastroenterology, Saitama Medical Center, Jichi Medical University, Saitama, JPN; 2 Department of General Medicine, Saitama Medical Center, Jichi Medical University, Saitama, JPN

**Keywords:** image-guided biopsy, acquired immunodeficiency syndrome, tumor, mycobacterial infection, immunoreconstitution inflammatory syndrome

## Abstract

Immunoreconstitution inflammatory syndrome (IRIS) was reported to occur in 7-13% of AIDS patients on anti-retroviral therapy (ART). IRIS due to Mycobacterium infection is one of the most difficult IRIS types to manage.

A male patient in his early 70s was diagnosed with AIDS and treated with an ART. One year after starting ART, abdominal ultrasound was performed for screening and a 4 cm hypoechoic mass was found from the outside of the stomach to the surface of the hepatic lateral segment. Based on various imaging tests, including contrast CT, a malignant tumor, such as malignant lymphoma, was suspected. Then, a percutaneous tumor biopsy was performed. Pathologically, the tumor was recognized as mycobacterial granulomas. Disseminated mycobacterium avium complex can produce granulomas anywhere in the body. The patient was diagnosed with a mycobacterial infection associated with IRIS.

When an intra-abdominal mass is found in a patient with HIV, both malignancy and mass formation due to opportunistic infections should be considered differential diseases.

## Introduction

Acquired immunodeficiency syndrome (AIDS) is associated with malignant tumors and malignant lymphomas. In patients with AIDS, infections rarely cause intra-abdominal mass formation. After starting an anti-retroviral therapy (ART) that is an anti-HIV therapy for patients with AIDS, various diseases, including opportunistic infections, have become apparent despite the effective treatment; this is called immunoreconstitution inflammatory syndrome (IRIS). Mycobacterium avium complex (MAC) infections, cryptococcal infections, and herpes zoster and cytomegalovirus infections have been reported as the main infections [1]. We report a case of a patient who was diagnosed with IRIS-associated mycobacterial infection due to an extramural mass during treatment of AIDS with ART.

## Case presentation

A male patient in his early 70s was diagnosed with AIDS one year ago because he had 14 CD4+ cells/μL and cytomegalovirus infection. The patient was started on ART after blood tests showed no cryptococcal infection, syphilis, or hepatitis A, B, and C. Chest CT showed no pulmonary tuberculosis. After starting the ART, renal damage appeared with a serum creatinine level of 1.31 mg/dL. Abdominal ultrasound was performed for screening, and a 4-cm hypoechoic mass was found from the outside of the stomach to the surface of the hepatic lateral segment, which was not present before the ART. Then, the patient was referred to our department of gastroenterology. He took a fixed-dose combination of bictegravir/emtricitabine/tenofovir alafenamide as the ART and trimethoprim-sulfamethoxazole combination medication.

The patient has not been drinking and had a smoking history of 60 pack-year and unspecified heterosexual intercourse. On admission, physical examination showed him to be alert and oriented, with a body temperature of 36.7°C, blood pressure of 136/87 mmHg, pulse rate of 67/min, and respiratory rate of 16/min. Left submandibular gland swelling was noted, but there were no hepatomegaly and palpable superficial lymph nodes.

Peripheral blood counts revealed a white blood cell count of 4550/μL and a reduced CD4+ cell count of 81/μL (Table 1). Blood chemistry tests were normal, except for alkaline phosphatase (ALP; 337 U/L), blood urea nitrogen (BUN; 25 mg/dL), and C-reactive protein (CRP; (0.17 mg/dL), which were mildly elevated. For infectious diseases, HIV-1 RNA (<20 copies/μL) and β-D glucan (7.0 pg/mL) were quantified. Mycobacterium tuberculosis-specific IFN-γ, anti-MAC antibodies, hepatitis B surface (HBs) antigens, HBs antibodies, and hepatitis C virus (HCV) antibodies were all negative. The tumor marker sIL-2R was mildly elevated to 714 U/mL. The tumor markers of alpha-fetoprotein (AFP), protein induced by vitamin K absence-II (PIVKA-II), ferritin, carcinoembryonic antigen (CEA), and carbohydrate antigen 19-9 (CA19-9) were all within the standard values.

**Table 1:** Laboratory findings WBC, white blood cell; PT-INR, prothrombin time-international normalized ratio; AST, aspartate aminotransferase; ALT, alanine aminotransferase; LDH, lactate dehydrogenase; ALP, alkaline phosphatase; GGT, gamma-glutamyl transferase; CRP, C-reactive protein; BUN, blood urea nitrogen; IFN, interferon; MAC, Mycobacterium avium complex; HBs, hepatitis B surface; HCV, hepatitis C virus; sIL-2R, serum interleukin 2 receptor; AFP, alpha-fetoprotein; DCP, des-c-carboxy prothrombin; CEA, carcinoembryonic antigen; CA19-9, carbohydrate antigen 19-9.

Test item	Reference Range	Value	Unit
WBC	3900-9800	4550	/μL
Neutrophil	40-74	59.2	%
Lymphocytes	19-48	28.4	%
CD4+ cell	344-1289	81	/μL
CD8+ cell	110-1066	749	/μL
Hemoglobin	12-17.6	13.7	g/dL
Platelet	13-36.9	21.9	10^4^/μL
PT-INR	0.9-1.2	0.99	
Albumin	4.1-5.1	4.1	g/dL
Total bilirubin	0.05-0.23	0.19	mg/dL
AST	13-30	21	U/L
ALT	10-42	14	U/L
LDH	124-222	178	U/L
ALP	106-322	337	U/L
GGT	13-64	19	U/L
CRP	0-0.14	0.17	mg/dL
BUN	8-20	25	mg/dL
Creatinine	0.65-1.07	1.02	mg/dL
Tuberculosis-specific IFN-γ	(-)	(-)	
Anti-MAC antibody	< 0.7	< 0.5	U/mL
β-D glucan	≦20	7	pg/mL
HIV-1 RNA	not detected	< 20	copies/μL
HBs antigen	(-)	(-)	
HBs antibody	(-)	(-)	
HCV antibody	(-)	(-)	
sIL-2R	157-474	714	U/mL
AFP	0-20	2	ng/mL
DCP	9-28	18	ng/mL
Ferritin	25-280	25.6	ng/mL
CEA	0-5	1.9	ng/mL
CA19-9	0-37	15.5	ng/mL

**Figure 1:**
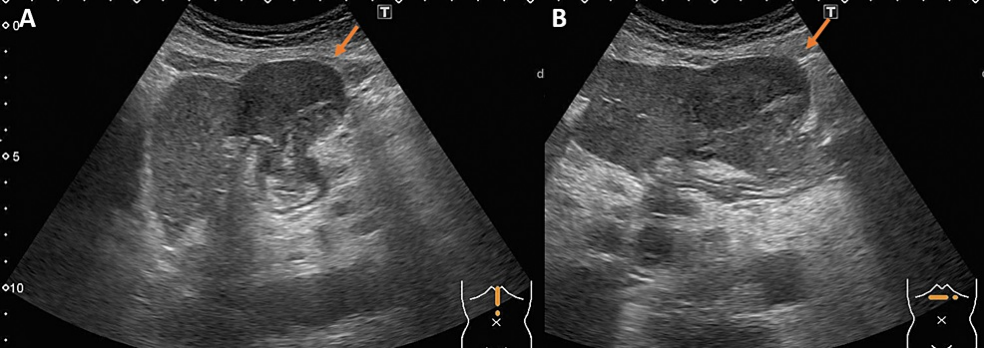
Abdominal ultrasonography (A) longitudinal axis, (B) horizontal axis A 4.5-cm, internally heterogeneous, hypoechoic mass is contiguous from the stomach to the liver.

**Figure 2:**
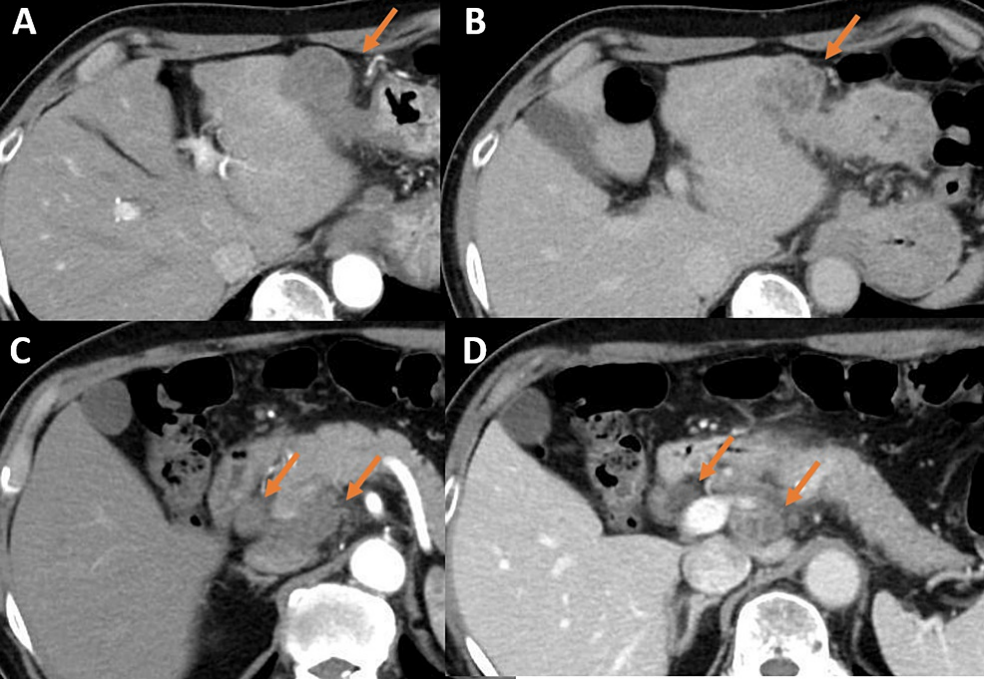
Abdominal contrast-enhanced CT An internal low-concentration mass between the liver and stomach in the arterial phase (A) and the delayed phase (B). Enlarged intra-abdominal lymph nodes around the portal vein in the arterial phase (C) and the delayed phase (D).

**Figure 3:**
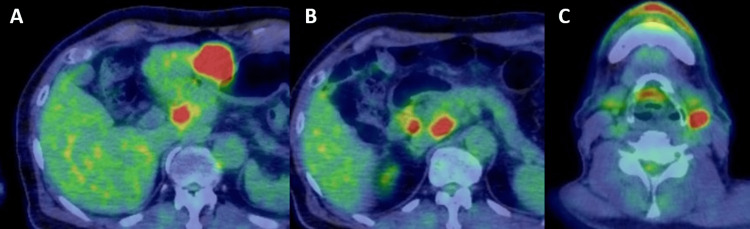
FDG-PET-CT FDG accumulation in (A) the mass between the liver and stomach, (B) intra-abdominal lymph nodes, and (C) left submandibular gland. FDG, fluorodeoxyglucose; PET-CT, positron emission tomography-computed tomography

Abdominal ultrasonography showed a 4.5 cm hypoechoic mass with a heterogeneous interior extending from the stomach to the liver (Figures 1A, 1B), and computed tomography (CT) showed an internal low-concentration mass at the same location and enlarged intra-abdominal lymph nodes (Figures 2A-2D). In addition, the left submandibular gland was enlarged, but there were no abnormal shadows in the lung fields. Positron emission tomography (PET)-CT showed an increased accumulation in the known mass, enlarged intra-abdominal lymph nodes, and left submandibular gland. The mass had a standardized uptake value (SUV) max of 9.83, and the enlarged intra-abdominal lymph nodes had an SUV max of 8.04 (Figures 3A-3C). There was no accumulation in the lungs or intestinal tract. Esophagogastroduodenoscopy showed no extramural indentation or mass exposure in the stomach, and colonoscopy showed no ulcer, tumor, or other inflammatory findings.

**Figure 4:**
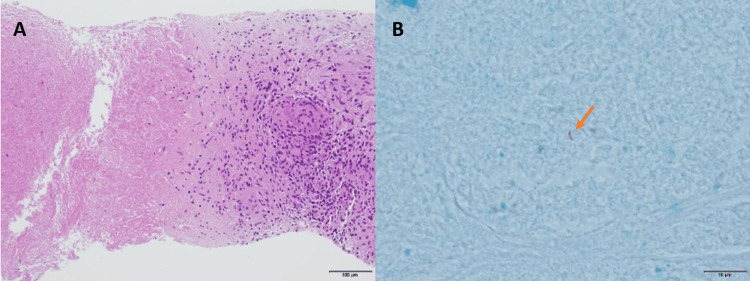
Pathological findings of the liver biopsy (A) An epithelial cell granuloma with caseous necrosis on hematoxylin-eosin staining (x40). (B) A single positive rod (arrow) on Ziehl-Neelsen staining (x400).

Percutaneous echo-guided tumor biopsy was performed by differential diagnosis including malignant lymphoma, gastrointestinal stromal tumor, metastatic tumor, poorly differentiated hepatocellular carcinoma, and intrahepatic cholangiocarcinoma. The patient was discharged without any complications. Based on pathological findings, hematoxylin-eosin staining showed epithelioid cell granuloma with caseous necrosis while Ziehl-Neelsen staining showed one positive rod (Figures 4A, 4B). No findings were suggestive of malignant lymphoma or malignant tumor. Based on the images, malignant tumors were considered first, but these were diagnosed pathologically as mycobacterial granulomas.

Then, a CT-guided tumor biopsy was performed, and a sufficient amount of sample was collected. Mycobacterium culture and polymerase chain reaction (PCR) for Mycobacterium tuberculosis and MAC were submitted, but the results were negative for staining, culture, and PCR, so the bacterial species was not identified. The image-guided biopsy could avoid the need for surgical exploration or resection in this case.

Treatment and outcome

**Figure 5:**
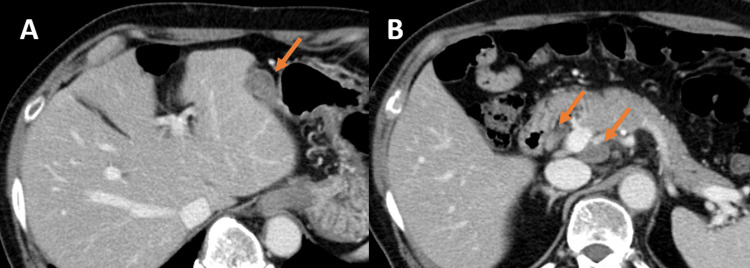
Abdominal contrast-enhanced CT after treatment The CT after five months of treatment showed that (A) the mass between the liver and stomach had shrunk to <2 cm, and (B) the intra-abdominal lymph nodes had also shrunk.

We judged that it would be difficult to examine the patient further, so the treatment was started with 1000 mg clarithromycin, 1000 mg ethambutol, and 500 mg rifabutin/day to target MAC, which is epidemiologically the most frequent. The treatment was continued without any complications, and the CT after five months of treatment showed that the mass had shrunk to <2 cm, and the intra-abdominal lymph nodes had also shrunk (Figures 5A, 5B).

## Discussion

In this case, an intra-abdominal mass was found during the treatment of AIDS, and a biopsy was performed to differentiate malignant tumors, such as malignant lymphoma, which turned out to be a granuloma caused by Mycobacterium. Kaposi’s sarcoma, non-Hodgkin’s lymphoma, and invasive cervical cancer are frequently seen in patients with AIDS and are included in the AIDS index malignancies. HIV-infected individuals are reported to be 1.44 times more likely to develop esophageal cancer, 1.69 times more likely to develop gastric cancer, 2.7 times more likely to develop liver cancer, and 7.3 times more likely to develop non-Hodgkin’s lymphoma than the general population [2,3]. It was reported that the causes of the disease are immunodeficiency, activation of oncogenic genes, other effects of HIV itself, and co-infection with oncogenic viruses [4]. We first suspected a malignant tumor because the patient had no symptoms of infection such as fever or local symptoms and a tumor had formed.

Although the number of newly reported HIV infections has remained constant in recent years, the number of patients with cumulative HIV/AIDS is increasing, and treatment with ART is started at the time of diagnosis. In a meta-analysis of 54 cohort studies, 1699 (13%) of 13103 patients who started anti-HIV treatment developed IRIS [5]. A study by the Japanese Ministry of Health, Labor, and Welfare reported that IRIS occurred in 7.1% of patients on ART in Japan from 2012 to 2016 [6]. About 20% of IRIS patients have been reported to develop tuberculosis and nontuberculous mycobacteria (NTM). Regarding opportunistic infections and HIV, the risk of developing tuberculosis is about 10 times higher in patients with HIV than in healthy adults due to the disruption of cellular immunity [7]. Furthermore, the risk of developing NTM is also thought to be higher. Many nontuberculous mycobacteria isolated from HIV infection have been reported. MAC is the most common cause of NTM infections associated with HIV infection, accounting for 64.0%-96.1% of the cases [7-9]. Mycobacterium (M.) kansasii was reported to be the second most common cause in Japan, but its frequency was < 10% [8].

MAC disease associated with IRIS causes local infections, such as cervical or intra-abdominal lymphadenitis, pneumonia, pericarditis, skin and soft tissue abscesses, vulvar ulcers, and central nervous system infections, in patients who have been started on ART and are responding to the treatment [10]. The route of infection of disseminated MAC was reported to be through the gastrointestinal tract and respiratory tract. Anemia, elevated ALP level, elevated lactate dehydrogenase (LDH level, hepatomegaly, splenomegaly, and lymphadenopathy may occur in MAC disease [11,12]. MAC antibody levels are often low in immunocompromised or immunosuppressed patients, such as patients with HIV [13], so the diagnosis is generally made by detecting the bacterial species by tissue culture. If a patient on ART develops disseminated MAC, ART should be continued [10].

In this case, the pathogen was not identified from various culture tests, and the route of infection was unknown because no significant findings were observed during the endoscopy. Despite the efficacy of ART, the mass was invasive and growing, so we diagnosed it as disseminated NTM disease associated with IRIS. Assuming MAC as the pathogenic bacteria, which is epidemiologically the most frequent in NTM infection, treatment was started. The basic treatment was clarithromycin or azithromycin plus ethambutol. In the present case, ART was continued, but the CD4+ cell count did not recover well, so rifabutin was added to the two drugs.

If the pathogenic bacterial species were identified, the optimal therapeutic agent would have been selected, including the duration of administration. Mycobacterium forms granulomas, which are histologically classified into exudative and proliferative reactions. In a report that applied this classification to pulmonary MAC, more bacteria were found in macrophages in cavitary and exudative lesions, but the number of bacteria in granular seeds tends to be lower, as the lesions shifted to proliferative reactions [14]. Although this case was not a case of pulmonary MAC, a similar condition was assumed. Granulomas might form in the abdominal cavity during the recovery of the immunity during ART, which may correspond to the proliferative reaction phase. Re-biopsy was performed, and it showed that the granulomas progressed to proliferative lesions, and the number of bacteria decreased further, which may have prevented the identification of the species. Although the antimicrobial therapy was performed in a situation where the species and susceptibility of the bacteria were unknown, both the images and data were improved. Therefore, the causative bacterial species could be MAC.

A similar case of an enlarged lymph node in the abdominal cavity after the initiation of ART has also been reported. PET-CT showed accumulation, and AIDS-related lymphoma was suspected. However, the pathological diagnosis was MAC based on a laparoscopic biopsy [15]. In MAC infections, macrophages are activated, and the FDG uptake in granulosa species is enhanced, which may make it difficult to distinguish it from cancer, including lymphoma. Therefore, pathology and culture results from the biopsy are very important.

## Conclusions

In this case, the patient was under ART for AIDS; however, Mycobacterial infection due to IRIS could not be suspected as the differential diagnosis for the mass at first. Disseminated MAC can produce granulomas anywhere in the body. For an early diagnosis and therapeutic intervention, infectious disease non-specialists also need to consider Mycobacterium infection as a differential diagnosis for tumors in patients with HIV/AIDS.
